# The Use of Acellular Dermal Matrix for Coverage of Exposed Joint and Extensor Mechanism in Thermally Injured Patients With Few Options

**Published:** 2008-06-24

**Authors:** Dhaval Bhavsar, Mayer Tenenhaus

**Affiliations:** Division of Plastic Surgery, UCSD Medical Center, Hillcrest, San Diego, CA

## Abstract

**Introduction:** One of the most devastating complications of deep burn injuries to the hand and finger is the exposure of joint, tendon, and neurovascular structures. The inevitable consequence of such injuries is severe deformity, often requiring joint fusions and digital amputations. Complicating this scenario is the anatomic limitation of few local and reliable soft tissue flaps available for this intricate distal distribution. This is particularly true for the patient who has suffered very large and deep thermal injuries. **Methods:** This series of cases describes the use of thin and meshed acellular dermal matrix to cover the exposed joint, tendon, and neurovascular structures, which resulted from severe thermal injuries. Securing the position of the lateral tendinous bands is a key component of the reconstruction. Composite staged reconstructions with either autologus split thickness skin graft or Integra provided definitive soft tissue coverage. Digits and joints were gently ranged when the overlying skin graft or Integra was adherent. **Results:** Of 26 digits treated in 4 patients, 19 digits demonstrated supple and durable skin coverage with acceptable joint mobility. One digit had to be amputated because of infection. Four digits developed Boutonniere deformity. Three digits underwent joint fusion at proximal interphalangeal joint. **Conclusions:** Early flap coverage, whenever possible, remains our preferred method of treatment of exposed joint, tendon, and neurovascular structures. When flaps are not feasible and faced with potentially salvageable yet terribly injured hands and fingers with complicated exposure, thin and meshed acellular dermal matrix may provide durable and vascularized soft tissue coverage while minimizing eventual deformities.

One of the most devastating complications of deep thermal injuries to the hand and fingers is the exposure of joints, tendons, and neurovascular structures, the consequence of which generally results in severe deformity often requiring joint fusions and digital amputations. This is particularly true for the patient who has suffered very large distribution and deep thermal injuries where few reliable and large soft tissue flaps are available for this intricate distribution. The standard of care for the management of exposed tendon, neurovascular, and joint structures is prompt coverage with well-vascularized tissue.^1^

Alloderm (Life Cell Inc, Branchburg, NJ) is a commercially available acellular dermis derived from human skin. The acellular nature of the product, as in several biosynthetic products, allows it to biointegrate in wound bed sites that might not be considered ideal for thicker skin grafts. These products, when utilized in meticulously debrided and prepared wound sites, can vascularize from the periphery if dimensions are favorable. In this work we describe the management of 4 severely injured burn patients in whom no locally available, well-vascularized soft tissue coverage was readily available. In all of these cases, as a result of the acuity and severity of the systemic injuries, free microvascular transfers were considered unsafe.

## MATERIAL AND METHOD

**Figure 1:**
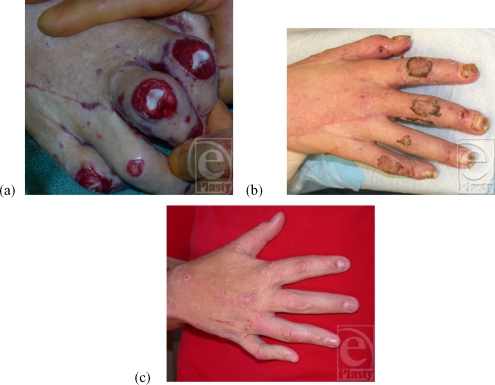
Patient no. 2: (a) Exposed PIP joints on right hand, (b) postoperative recovery—day 21 after alloderm placement and split thickness skin graft application and (c) follow-up after 9 months.

**Figure 2:**
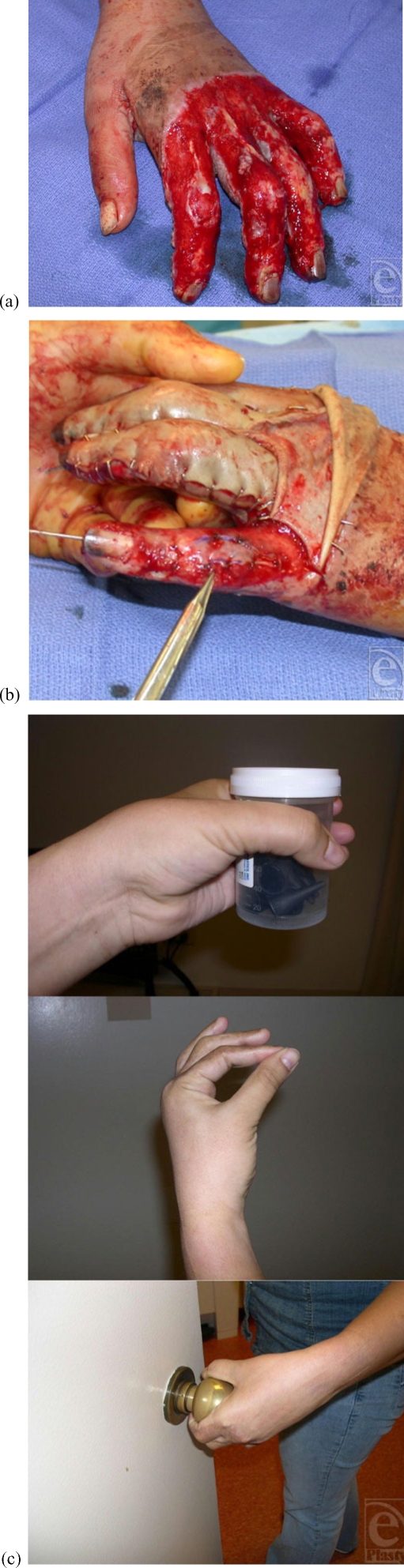
Patient no. 4: (a) Deep burn of the left hand with exposed PIP joints and extensor mechanism loss of ulnar 3 digits, (b) alloderm application on PIP joint of left little finger, all exposed joints were treated in a similar way, and (c) follow-up at 18 months.

Between years 2003 and 2005, we encountered 4 patients with full-thickness burn injury to either one or both the hands. Following burn wound debridement, proximal interphalangeal joints were exposed and extensor mechanisms over the joint area in these patients were partially destroyed (Figure 1a and 2a). Three of the four patients had large distribution of burn injury and all 4 were acutely ill which precluded intervention with local or remote skin flaps or microvascular reconstruction. We chose acellular dermal matrix (ADM) to cover the exposed joint, tendon, and neurovascular structures. Except for the first case, where nonmeshed ADM of average intermediate thickness was used, we preferably used meshed and thin ADM to cover the defects. The pieces of ADM were trimmed to match the size of defect in a way that there would be an overlap of few millimeters to assure vascularization from healthy margins (Figure 2b).

In each case, the ADM was secured with a combination of absorbable sutures and a dilute thrombin glue spray. The position of lateral tendinous bands was secured bilaterally with ADM in an effort to minimize subsequent displacement and deformity. Allograft was used to overlay the ADM as a provisional coverage in 3 of the 4 cases, whereas Integra (Integra Life Sciences, Plainsboro, NJ) provided the basis for soft tissue coverage in the forth case. The allograft covered subsets were maintained in dilute Sulfamylon solution moistened dressing, whereas the Integra covered case was affixed with negative pressure therapy (VAC; Kinetic Concepts Inc, San Antonio, Tex) and an Acticoat (Smith Nephew Wound Care, Largo, Fla) dressing. Fingers and joints were gently ranged when the overlying coverage demonstrated adherence. At approximately 2 weeks postapplication of the ADM, a thin and non—meshed split thickness skin graft was applied. The details of the patient demography and wound defects are listed in Table 1.

## RESULTS

Acellular dermal matrix integrated successfully in most of the treated sites. The resultant biointegration enabled staged, definitive, and durable soft tissue coverage. All but one, patient achieved functional ability in treated hands and returned to their work. Patient no. 1, who had more severe burn injury and lost 3 digits, could not return to original work and had to undergo occupational rehabilitation. Figure 1c, 1d, and 2c represent reconstructive outcomes in patient 2 and 4, respectively. Of 26 treated digits, 19 digits resulted in supple and healthy skin coverage. The passive range of movement was evaluated for patients 2 and 4 (Table 2). There was minimal restrictive scar formation over the treated joint area and extensor mechanism was in balance in these digits. One of the treated digits had to be amputated at the level of proximal phalanx because of uncontrolled infection during the immediate postoperative period. Boutonniere deformity was observed in 4 digits, mostly affecting little finger. One of the patients (patient no. 2) refused physical therapy and did not attend the follow-up clinic. This patient presented with unstable joints, interfering with function, after 9 months. We performed joint fusion on these digits to provide arthrodesis in functional position. The flaps were successfully raised through ADM covered joint areas for access and they healed uneventfully. The complications are detailed in Table 1.

## DISCUSSION

Complex and extensive injuries to the hands and fingers in patients surviving devastating thermal injuries can prove extremely difficult to treat. Even when optimally treated, the expected resultant deformities challenge the best of surgeons and impair the quality of life for our patients. During the acute setting, tendons and nerves left open may desiccate, devitalize, and scar down with resultant loss of function. Open joints and exposed bone can similarly desiccate, colonize with bacteria, become infected, and lose their functional capacity. If left open, exposed vascular structures desiccate, thrombose, or lose integrity leading to aneurysm or hemorrhage. In complex functional structures like the hand or foot, the ensuing disabilities and deformities result in life-altering consequence. It is for these reasons that rapid attempts at soft tissue reconstruction and coverage are of paramount importance for functional and aesthetic recovery.

Skin grafts alone do not reliably cover exposed tendons that have lost their paratenon and bone denuded of its periosteum or open joints.^2^ Interestingly and despite the fact that thin split thickness skin grafts have demonstrated satisfactory take on the thinner flat bones of the face skeleton, scalp, and retro-orbita,^3^ attempts at skin grafting over complex wounds of the hand and fingers with open bone and joints generally fail.^4^,^5^ At present, well-vascularized flap coverage remains our best option and yet these reconstructive efforts can often prove quite challenging in the hemodynamically compromised patient with major and diffuse soft tissue deficits.

Integra, a biosynthetic dermal replacement material now commonly employed in acute and reconstructive burn care, has shown some capacity of biointegrating onto exposed bony surfaces such as the calvarium and over the anterior tibia,^6^–^8^ even when devoid of periosteum.

Alloderm, a commercially available cryopreserved acellular dermal matrix, is extensively employed in the fields of aesthetic surgery for soft tissue augmentation^9^ (ie, lip augmentation and nasal dorsum augmentation) as well as in reconstructive surgical applications like bladder slings,^8^,^10^ hernia repair,^11^,^12^ and breast reconstruction.^13^ The proprietary process results in a dermal substrate, which is devoid of cellular elements, while retaining network of dermal matrix and vascular channels. When placed in vivo, ADM biointegrates by cellular repopulation and revascularization. Clinical experience with the product has found it to be somewhat tolerant of biologic and bacterial load, as evident in several published clinical series of complex ventral hernia and parastomal repair.^12^,^14^

In the cases presented we hypothesized that these “product attributes” would, if successfully applied, facilitate the development of a vascularized bed necessary for definitive coverage. In so doing, critical structures would be immediately protected from exposure and desiccation. Allograft or Integra coverage over ADM, in combination with Sulfamylon dressings or the occlusive environment of the VAC, might then aid in the prevention of desiccation. Suturing strips of thin and meshed ADM to remnants of the extensor mechanism and lateral bands would hopefully prevent subluxation and migration of critical structural elements while effecting a soft and durable coverage so vital for the eventual reconstructive efforts that we knew were forthcoming.

Our experience with these difficult cases has led us to the following recommendations with regards to the management of these complex, thermally injured hands and fingers with open joint, and the loss of segments of the extensor mechanism. Escharotomies, early judicious excision, optimal resuscitation, and tissue-protective strategies constitute the mainstays of therapy. Every attempt is made to optimize blood flow to the ischemic tissues, minimizing edema with extremity elevation, optimizing effective resuscitative volumes, and efforts while providing prompt vascularized coverage whenever possible. The management of biologic and bacterial burden is of critical importance and affected by judicious and expeditious debridement. Every effort should be made to protect salvageable tissues and structures in an attempt to minimize further injury and maintain an infrastructure critical for reconstruction. Moist antimicrobial dressings may aid in prevention of desiccation while providing a measure of microbial control. Allograft coverage can similarly provide biologic protection against desiccation while promoting fibrovascular ingrowth of tissue. The negative pressure wound therapy devices hold great promise in the management of many complex wounds. Clinical experiences have suggested that this modality might be beneficial in reducing edema while affording protective splinting. Others have suggested that these devices may improve vascularization when applied over grafts and biosynthetics increasing local granulation tissue development.^15^–^19^

The most common and traditional sources of soft tissue coverage for the hand and fingers consist of cross finger flap, fan flap, radial forearm flap, dorsal interosseous flap, groin flap, abdominal, and sub mammary cutaneous flaps as well as free microvascular transfers. Whenever possible, we prefer to employ these modalities early in treatment. When these soft tissue coverage options are not available or safe then we advocate an attempt at coverage with thin and meshed ADM. We have found that thinner and smaller segments are accepted most reliably and we advocate fixing the ADM to the residual extensor segments and lateral bands when segmental discontinuity exists.

Although larger and thicker sheets of ADM have proven very beneficial in later reconstructive applications, successful engraftment and incorporation of these larger sheets of unmeshed ADM in the patients with severe, diffuse, and acute thermal injury presentations have been difficult to accomplish. In all of the cases presented, we could not reliably identify locally available tissues of sufficient quality and extent to afford local coverage. Furthermore, our experience has shown us that without some attempt at maintaining the relative location of the residual musculotendinous structural support mechanism, very significant deformities seemed a likely and expected consequence. The ADM in this setting does not replace or function as an extensor tendon per se. Rather; it provides stable and durable coverage while helping prevent the abnormal migration of residual musculotendinous structures that lend themselves to significant and complex deformity. Subsequent reconstructions like joint fusions or tendon reconstructions are, as a result, facilitated.

When faced with frank necrosis, forth-degree burns, or wet gangrene, the surgical course is easily established. In cases such as those presented in this series, the timing of definitive joint fusion or even amputation for expected deformity and disability is often difficult to establish during the early acute interventions. Neighboring and surrounding soft tissues are generally also involved in the thermal insult, edematous and fragile making attempts at primary bone shortening, local flap development, and the use of implanted foreign materials imprudent.

As with all the biologic and biosynthetics employed in soft tissue reconstructions, it is very important to gain a familiarity with the characteristics and peculiarities of their biointegration. In the early healing phase, 7 to 20 days postoperatively, epithelialization of this composite tissue can appear almost multifocal in nature. This is a well-known phenomenon identified by numerous practitioners using ADM in larger burn release reconstructions. Some experience by the clinician and staff is needed to appreciate the stages of revascularization and epithelialization as it proceeds in this setting.

Our experience with early-complex-composite reconstruction of the locally and thermally injured tendon and soft tissues of the dorsal hand has been promising. Ideally, it would be wonderful to provide not only vascularized soft tissue coverage but also a functional musculotendinous reconstruction to the fingers as well. The consequences of hemodynamic, anesthetic, and logistical concerns certainly complicate and compromise the early course of the patient with life-threatening burns. Life over limb reality remains paramount. As a result, we have tried to develop procedures like these that add little extra to the overall operative time and yet seem to afford a degree of structural protection for the patient. In doing so, we feel that we are facilitating future reconstructive possibilities for our patients by providing and maintaining a reliable infrastructure and soft tissue coverage that is often the rate limiting step in our patients' inevitable future reconstructive needs.

Despite the fact that 2 of the 4 patients presented were lost to follow-up shortly after discharge, we strongly advocate an early and comprehensive rehabilitative regimen to closely monitor patient-related outcomes while optimizing desired functional outcomes.

## CONCLUSION

Early flap coverage, judicious use of splinting, gentle range of motion, and subsequent strengthening regimens, whenever possible, remain our preferred method of treatment for exposed joint, tendon, and neurovascular structures. When flaps are not feasible and faced with potentially salvageable yet terribly injured hands and fingers with complicated exposure, thin and meshed ADM may facilitate and provide durable and vascularized soft tissue coverage while minimizing eventual deformities.

## Figures and Tables

**Table 1:** Patient Data*

				No. of digits with exposed joints				
Patient no.	Sex/age, y	%, TBSA	Etiology	Right (rt)	Left (lt)	Type of ADM used	Provisional secondary coverage	Complications
1	M/27	87	Self-immolation with volatile fuel	4	4	Nonmeshed, rectangle	Allograft + Sulfamylon	Amputation of lt middle finger; Boutonniere's deformity of little finger on lt hand
2	M/32	82	MVA and flaming vehicle	4	3	Meshed, thin, ellipse	Allograft + Sulfamylon	Ulnar 3 digits on lt hand underwent joint fusion; Boutonnière's deformity of little and ring fingers on rt hand
3	F/54	79	House fire	4	4	Meshed, thin, ellipse	Allograft + Sulfamylon	Boutonniere's deformity of little finger on rt hand, mild flexion deformity of PIP joints of all 4 fingers on lt hand
4	F/27	62	Flame	0	3	Meshed, thin, ellipse	Integra + Acticoat + VAC	None

* TBSA, total burn surface area; ADM, acellular dermal matrix; MVA, motor vehicle accident.

**Table 2:** Passive range of motion (flexion)*

Patient	MP (90^−^)	PIP (110^−^)	DIP (65^−^)
2 (Right hand)	80–90	80–100	50–60
2 (Left hand)	70–90	20–80	40–50
4 (Left hand)	90	100	60

* MP indicates metacarpophalangeal; PIP, proximal interphalangeal; DIP, distal interphalangeal.
